# Exploring infant and young child‐feeding practices among mothers of well‐nourished children in northern Ghana: An INPreP substudy

**DOI:** 10.1002/fsn3.3800

**Published:** 2023-11-20

**Authors:** Edith Dambayi, Emmanuel Nakua, Raymond A. Aborigo, Samuel T. Chatio, Maxwell A. Dalaba, Enos Sekwo, James K. Adoctor, Winfred Ofosu, Abraham R. Oduro, Engelbert A. Nonterah, the INPreP Study Team

**Affiliations:** ^1^ Navrongo Health Research Centre, Social Science Department Ghana Health Service Navrongo Ghana; ^2^ Department of Epidemiology and Biostatistics Kwame Nkrumah University of Science and Technology Kumasi Ghana; ^3^ Institute of Health Research University of Health and Allied Sciences Ho Ghana; ^4^ Upper East Regional Health Directorate Ghana Health Service, PMB Bolgatanga Ghana; ^5^ Research and Development Division Ghana Health Service Accra Ghana; ^6^ Julius Global Health, Julius Center for Primary Care and Health Sciences University Medical Center Utrecht, Utrecht University Utrecht The Netherlands

**Keywords:** community practices, first 1000 days, Ghana, infant and young child feeding (IYCF), nutrition

## Abstract

This study investigated infant and young child‐feeding (IYCF) practices among mothers of well‐nourished children in northern Ghana. This was a qualitative study where in‐depth individual interviews were conducted with participants. The interviews were audio recorded, transcribed, and QSR Nvivo software version 11 was used to organize the data before thematic analysis. It was observed that mothers of well‐nourished children were likely to adhere to breastfeeding guidelines and also practice appropriate complementary feeding. Furthermore, these mothers mostly had some form of support from their husbands and mother‐in‐laws in feeding their infants. While adoption and adherence to appropriate IYCF practices contribute to improved nutrition outcomes in children, social support systems are needed to sustain the practice.

## Graphical abstract

Child undernutrition affects growth, impairs cognition and leads to poor educational performance with low economic productivity in adulthood. Appropriate Infant and Young Child Feeding (IYCF) practices such as 6 months exclusive breastfeeding, followed by adequate and safe complementary feeding up to 2 years is essential in preventing child undernutrition.

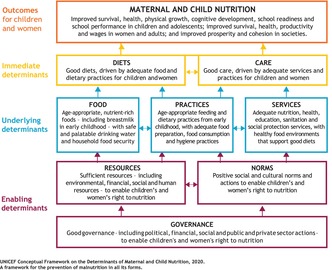

## 1 INTRODUCTION

The Global Nutrition Report shows that 22% of children under 5 years of age are affected by stunting, while 6.7% are affected by wasting. In the Western Africa sub regions, the prevalence of stunting is 30.9%, which is higher than the global average of 22.0%, and the prevalence of wasting, which is 6.9%, is also higher than the global average of 6.7% (Global Nutrition Report, 2022).

It has been demonstrated that key factors contributing to child malnutrition in Sub‐Sahara Africa include poverty, early weaning, a shorter duration of exclusive breastfeeding, poor feeding, bottle feeding, and limited consumption of fruits (Bain et al., 2013). Furthermore, sociocultural practices, climatic conditions such as droughts, poor soils, and deforestation have also been reported as factors affecting the nutritional status of children, especially in SSA (Quamme & Iversen, 2022).

In Ghana, there has been a gradual improvement in the nutritional status of children, yet malnutrition remains prevalent. In the 2008 Ghana Demographic and Health Survey (GDHS), about 3 in 10 (28%) of Ghanaian children were chronically malnourished or stunted (Ghana Statistical Service & Ghana Health Service, 2009). This experienced a modest reduction in 2021, with 18% of children under 5 years of age affected by stunting, while 6% of children under 5 years of age affected by wasting (Ghana Statistical Service, 2022).

Moreover, it has been reported that malnutrition is one of the leading causes of mortalities among children under 5 years in the Kassena‐Nankana Municipality (Babayara & Addo, 2018).

The consequences of undernutrition for children under 5 years have been well researched and documented (Glewwe & Jacoby, 2016). The results of early undernutrition for children include suboptimal brain development, which negatively affects cognitive development, educational performance, and economic productivity in adulthood. There is also an increased risk of infection, death, and delayed cognitive development, leading to low adult income levels, poor economic growth, and intergenerational transmission of poverty (De & Chattopadhyay, 2019). Children with undernutrition have weak immunity and are more likely to die from common diseases such as malaria, respiratory infections, and diarrheal diseases (Fischer Walker et al., 2013; Olusanya et al., 2018).

Appropriate infant and young child‐feeding (IYCF) practices are essential to preventing child undernutrition. This approach involves exclusive breastfeeding for 6 months, nutritionally adequate and safe complementary feeding starting from the age of 6 months, and continued breastfeeding up to 2 years of age or beyond (World Health Organization & United Nations Children's Fund (UNICEF), 2003).

Developing agencies such as the United Nations Children's Fund (UNICEF), the World Bank, and health researchers across various institutions have identified the causes of undernutrition to include the types of foods consumed; IYCF practices adopted; challenges faced by caregivers; and finally, the availability of support systems for caregivers (UNICEF and WHO, 2013).

In Africa, particularly in Ghana, mothers are the primary caregivers of children; they have significant control over factors critical for child well‐being, including food preparation and storage, feeding practices, psychosocial care, hygiene, health practices, and newborn care (Bimpong et al., 2020; Lartey, 2020; Nti & Lartey, 2008). Previous studies have shown that several challenges, including household chores, workload by mothers in the formal sector, pressures from close associates, especially grandmothers, to introduce prelacteal feeds to new‐born babies, low breastmilk, swollen breasts or sore nipples, and unavailable safe complementary feeds prevent mothers from adopting IYCF practices (Mukuria et al., 2016; Naah et al., 2019). Many times, the factors that support the nutritional outcome of children include the influence of social relations, access to safe, nutrient‐rich complementary foods, and adequate child healthcare (Branca et al., 2015; Mukuria et al., 2016).

The introduction of the Ghana national nutrition policy in 2016 is a step that was undertaken to implement nutrition programs and tackle issues of undernutrition in Ghana; yet child undernutrition still persists (UNICEF Ghana, 2016). In the policy, nutrition interventions such as early initiation of breastfeeding, exclusive breastfeeding, iron and folate supplementation (IFA), vitamin A supplementation (VAS), management of acute malnutrition (CMAM), and complementary feeding were implemented to address this problem. A follow up study was carried out on the implementation of these interventions, majors' bottlenecks were found which suggests reasons for the high prevalence of the problem. Service delivery was a major issue; this was linked to inadequate pre‐service training on breastfeeding for frontline workers, a lack of support for mothers to breastfeed in the first hour of birth, a poor staff attitude, social norms on feeding after delivery, and unstructured orientation on exclusive breastfeeding by frontline workers, among others (Yawson et al., 2017).

Several studies have reported key factors contributing to high undernutrition among children, especially in SSA. Furthermore, most studies have used quantitative methods to establish factors contributing to this problem (Bain et al., 2013; Id et al., 2019; Tette, Sifah, Nartey, et al., 2016; Tette, Sifah, Tete‐Donkor, et al., 2016; van Cooten et al., 2019). A few qualitative studies have also been conducted among mothers of well‐nourished children and mothers of malnourished children (Cheah et al., 2007; Kismul et al., 2015; Reiher & Mohammadnezhad, 2019). The focus of previous qualitative studies was centered on social factors and maternal profiles; however, for this particular study, the team used the global strategy for IYCF practices as a guide to explore this area. An additional unique feature of the current study is that the study team included only well‐nourished children, which has not been investigated by previous studies. The inclusion of well‐nourished children creates an opportunity to explore the enablers and strategies adopted by these mothers to ensure that their children are well‐nourished.

### 1.1 Key messages

1. Undernutrition is a major source of critical health and developmental challenges, especially among children in developing countries, including Sub‐Saharan Africa (SSA).
2. The identified causes of child undernutrition include the types of foods consumed, IYCF practices adopted, challenges faced by caregivers, and the availability of support systems for caregivers.
3. Appropriate IYCF practices improve the nutritional status of children.
4. This paper adds to the literature showing that mothers of well‐nourished children are likely to adopt IYCF practices.
5. The successful implementation of IYCF practices is also dependent on social support systems.
6. We recommend that future research should involve a comparison of IYCF practices between well‐nourished and malnourished children to understand the point of departure between the two groups.

## 2 METHODS

### 2.1 Study design

The study used a qualitative research method where in‐depth individual interviews (IDIs) were conducted with participants. Qualitative research assumes that “reality is subjective and multiple, as experienced by individuals participating in a study”. Qualitative research is “descriptive” of the process and the meanings gained through words. This approach helps in capturing the feelings and experiences of study participants on the issue under investigation (Creswell, 2014).

Therefore, this approach was considered appropriate because it has helped the study team gain an in‐depth understanding of the kinds of feeding practices and strategies adopted by mothers to improve the nutritional status of their children.

The study used community health planning and services (CHPS) compounds as a unit of recruitment for study participants. CHPS is one of the strategies for achieving universal health coverage, providing essential primary healthcare services, especially for women and children (Ministry of Health, 2017). The study was carried out as part of the broader INPreP (Improved Nutrition preconception, during pregnancy and post‐delivery) project (https://www.southampton.ac.uk/global‐health/research/lifecourse‐epidemiology/inprep/about‐us.page). The INPreP study is a research study that is focused on nutrition during preconception, conception, and after delivery. The study intends to make a case for why early nutrition is critical for maternal health and child survival, as well as the health outcomes of future generations.

### 2.2 Study area

The study was conducted in the Kassena‐Nankana East Municipal in the Upper East Region of Northern Ghana. The Municipal is home to the Navrongo Health Research Centre (NHRC). It has a total land area of about 1674 km^2^ of semi‐arid grassy scrubland and is located on Ghana's northern border with Burkina Faso. The dominant ethnic groups in the study area are the Kassena and the Nankana. The ecology of the area is more characteristic of the Sahelian heartland than regions in the southern part of the country. Subsistence agriculture represents the mainstay of the local economy; rainfall is limited from May to October, thus restricting cultivation to a single growing season. The major crops grown are millet, maize, sorghum, rice groundnuts, leafy vegetables, cowpeas, bambara beans, okra, tomatoes, and onions. Nutritional adversity is common, exacerbating the mortality impact of infectious disease in the municipality (Babayara & Addo, 2018). The study area is prone to food insecurity due to the climate conditions, which are characterized by the dry and wet seasons, and this has been reported to have a seasonal effect on the nutritional status of children in the area (Nonterah et al., 2022).

Kassena‐Nankana East municipal has an estimated population of 99,895 (Ghana Statistical Service, 2022). It has seven sub‐municipalities, one hospital, two health centers, 20 functional community‐based health planning and services facilities (CHPS compounds), and one Christian Health Association of Ghana (CHAG) facility. The Navrongo War Memorial hospital, a secondary health facility, serves as the major referral facility and also offers comprehensive maternal services (Nonterah et al., 2020). The Navrongo Health Research Centre (NHRC) also conducts high‐quality health and socio‐demographic surveillance in the area to inform health policy and generates sampling frames for various research activities (Oduro et al., 2012).

### 2.3 Study population

The study population constituted mothers with children between 24 and 59 months who were well‐nourished in the study area. Although IYCF practices involve exclusive breastfeeding for 6 months, nutritionally adequate and safe complementary feeding starting from the age of 6 months with continued breastfeeding up to 2 years of age or beyond, for this particular study, the team included only children within the 24–59 months to find out whether all these recommendations stated above were adopted by the mothers. Also, this group is at a higher risk of malnutrition.

The study used the World Health Organization's child growth standards to identify a well‐nourished child. This involves the use of mid‐upper arm circumference (MUAC) measurements and the age‐for‐height and age‐for‐weight measurement in the growth monitoring chart (standard deviation) to establish the nutritional status of a child. A well‐nourished child was defined as someone with MUAC ≥135 cm (green color on the MUAC tape). Also, children within −2 to +2 standard deviation for age‐for‐height and age‐for‐weight indicated a well‐nourished child.

Therefore, for this study, these three indicators were used to define a well‐nourished child and a child had to meet all three criteria to be qualified as well‐nourished.

The inclusion criteria included the following: Being Kasem or Nankam and residing in the selected zone for more than 2 years; must have a child between 24 and 59 months; and agreed to take part in the study and provide informed consent. In order to find out if there are any ethnic differences in sociocultural practices that may influence child nutrition, ethnicity was considered in the selection of study participants.

### 2.4 Sampling techniques

The Navrongo Health Research Centre runs a demographic surveillance system (NHDSS) within the Kassena‐Nankana East municipal and the Kassena‐Nankana West District. The catchment area of the NHDSS is divided into east, south, west, north, and central zones. The north and west zones are inhabited mostly by Kassena, while the east and south are predominated by Nankana ethno‐linguistic groups. The central zone hosts heterogeneous ethnic groups, which is not the interest of the study. Therefore, one zone representing the Kassem group was purposively selected, and one zone from the Nankam group was selected. These zones are located in the Kasena‐Nankan east municipal. In order to find out if there are any ethnic differences in sociocultural practices that may influence child nutrition, ethnicity was considered in the selection of study participants.

Purposive sampling is generally used in qualitative studies. This enables researchers to select study participants who will provide suitable information to help address the research objectives (Judith Green, 2014).

This study therefore purposively selected two CHPS compounds from the North and two CHPS compounds from the South zones, representing the Kasem and the Nankam ethno‐linguistic groups. Within the selected zones, we generated a list of well‐nourished children from the HDSS system.

Based on the list of well‐nourished children, their mothers were contacted through telephone calls and scheduled for the interviews. Additionally, community health volunteers in the selected CHPS zones helped identify the houses of the identified potential participants. Those who agreed to participate were interviewed.

### 2.5 Pre‐testing and data collection procedures

Experienced research assistants with extensive knowledge in qualitative data collection went through 2‐day training to familiarize with the study protocol and data collection procedures. Role‐play was done as part of the training to better understand how to ask the questions appropriately during the actual data collection. The Kassena‐Nankana West District, which is adjacent to the study district and has a relatively similar characteristics, was used for the pretesting of the study tools. Two pretests were conducted among mothers of well‐nourished children. The pretesting also helped the researchers assess response latency (the amount of time it takes to complete a survey), which was then reported in the introduction. This strategy was very important because it helped to assess whether the researcher and the respondent interpreted the survey in the same way.

Data were collected face to face in the form of IDIs while observing Covid‐19 prevention guidelines. Interviews were conducted in the two main local languages (Nankani or Kasem), audio‐recorded in the local language, and later translated and transcribed into English. The transcripts were reviewed for accuracy and completeness and imported into QSR NVivo 12 qualitative software for coding.

A structured questionnaire was used to elicit the socio‐demographic characteristics of the mothers, while an interview guide was used to elicit information on appropriate IYCF practices from the mothers/caregivers. Some of the broad questions asked were regarding IYCF practices and general support for child care.

### 2.6 Measures taken against COVID‐19 pandemic

Face masks and hand sanitizers were used during each household visit. Each study participant was given a face mask before an interview began. Between interviewer and respondent, there were two meters to prevent close contact. The audiotape recorder was sanitized after each interview.

### 2.7 Quality control

To ensure the validity of study findings, triangulation (data analysis involving several investigators in the analysis process) and reliability methods (Korstjens & Moser, 2018) were adopted. Reliability was checked using NVivo 12 qualitative software to ensure that coding consistency is always at least 80%. Also, to ensure the accuracy of the data, data collectors were fluent in the two local languages of the study area. Audiotape recorders were also assessed before using them on the field for data collection. In the process of triangulation, two independent researchers developed a code book using the original research questions. After analyzing the data, the researchers then merged the results to compare similarities.

### 2.8 Thematic saturation

Twenty IDIs were conducted with mothers of well‐nourished children. After each field visit, interviews were transcribed, and the team held discussions to find out if new ideas were emerging from the discussions. If there was the need to add question or emphasize certain points, then these were considered in the next interviews. The team agreed on data saturation for the 20 interviews when no new information or themes emerged from the data collected. This aligns with the definition of data saturation, which is said to occur when very little or no new phenomena emerge from the data so far (Guest et al., 2006).

### 2.9 Data analysis and presentation

The data were subjected to thematic analysis. Data management and coding were carried out in NVivo software version 12. Audio recordings from the in‐depth interviews were transcribed verbatim by two research assistants. Once transcribed, the transcripts were read repeatedly to check for consistency. A list of key statements, ideas, and opinions expressed on each topic of discussion was made. Topic codes were developed, which were informed by the interview guide. The final sets of codes were agreed upon by the team, and definitions for each code were developed. Each transcript was then coded, and additional codes were identified and added to the codebook. Some themes were pre‐set based on the interview guide, and some were generated from the data. The data was then presented in a narrative summary. The socio‐demographic characteristics of the respondents were described and presented in a table.

### 2.10 Ethical considerations

Ethical clearance was obtained from the Navrongo Health Research Centre Institutional Review Board and the Committee on Human Research, Publication and Ethics of KNUST. Administrative permission was also granted by the municipal director of health services for access to the child welfare records at the selected CHPS compounds and to interact with the community health nurses. Signed informed consent was obtained from each participant before study procedures were carried out.

## 3 RESULTS

### 3.1 Background information of participants

We conducted a total of 20 in‐depth interviews with mothers.

The mean maternal age was 30 (range: 20–42) years, with participants almost evenly distributed across the age groups. All 20 participants were married. Self‐employment was the dominant source of income (55%), with trading (50%) being the main economic activity among participants. The majority (70%) of participants had income levels below 1000 Ghana cedis ($164), with a further 20% reporting income levels between 2000 ($328) and 3000 ($492) Ghana cedis. One participant had no formal education, while 30% had completed secondary education. The majority of the participants (70%) had one child under five at the time of data collection (Table 1).

**TABLE 1:** Distribution of sociodemographic characteristics of study participants.

Characteristics	Category	Frequency (*N* = 20)	Percent (%)
Age group (years)	20–25	4	20.0
	26–30	5	25.0
	31–35	6	30.0
	36+	5	25.0
Marital status	Married	20	100.0
	Single	0	0.0
Occupation	Hairstylist	1	5.0
	Nurse	1	5.0
	Unemployed	3	15.0
	Seamstress	2	10.0
	Teacher	3	15.0
	Trader	10	50.0
Source of income	Self‐employed	11	55.0
	Housewife	5	25.0
	Government employed	4	20.0
Religion	Christianity	20	100.0
	Others	0	0.0
Highest level of education	No schooling	1	45.0
	Primary	3	5.0
	Junior High school	9	15.0
	Senior High School (SHS)	3	15.0
	Tertiary	4	20.0
Household income	Less than 1000	14	70.0
	1000–1999	1	5.0
	2000–3000	5	25.0
Number of children under 5 years	One	14	70.0
	≥ Two	6	30.0

### 3.2 Thematic presentation of results

**FIGURE 1:**
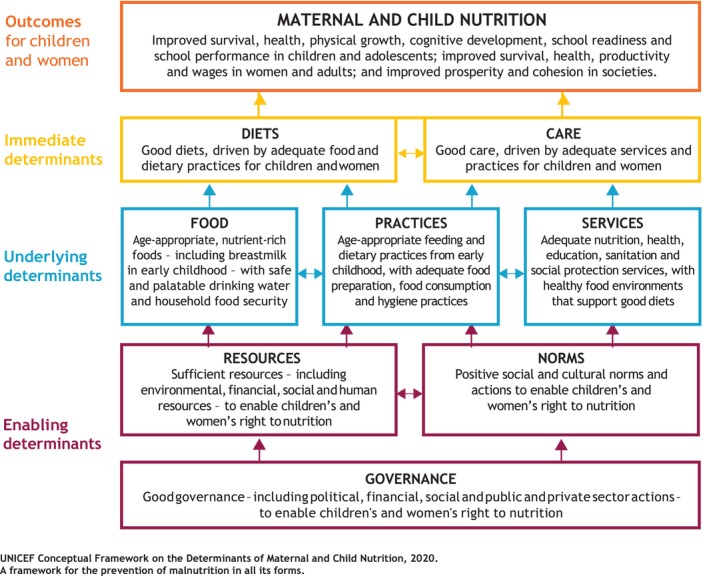
This study adapted UNICEF's conceptual frame on Maternal and Child Nutrition (UNICEF, 2021).

After thematic analyses, four important themes were observed in the data that explain the appropriate adoption of IYCF practices by the mothers. They include knowledge about breastfeeding (sub‐themes include initiation, duration, and frequency of breastfeeding), knowledge about complementary feeding, overcoming sociocultural barriers, and receiving support from husbands and mother‐in‐laws (Figure 1).

### 3.3 Knowledge about breastfeeding

#### 3.3.1 Initiation of breastfeeding

Many of the participants had fair knowledge about appropriate breastfeeding practices. Most mothers acknowledged the need for the early initiation of breastfeeding. According to the mothers, the first breast milk contains nutrients, which are good for newly born babies. They believed this could protect the babies from diseases and other infections. Mothers reported that they started breastfeeding immediately after delivery, largely because of the health education and support from the nurses. This quote illustrates the forgoing explanation.

They say that milk, they nurses have made us aware that, the milk that comes out after you have given birth, they say that milk is what helps the child, so when you give this milk, the baby won't be falling sick all the time. This would even help the child to grow well. (IDI, mother of a two‐year‐old child, north zone)

#### 3.3.2 Duration of breastfeeding

The sub‐theme on duration of breastfeeding is another essential component of breastfeeding that influences nutrition outcomes. Several participants reported that they breastfed for 2 years; a few of them breastfed for 1 year and 6 months. Work‐related activities such as trading and farming were reported as the main reason why they were unable to breastfeed their children for 2 years. They demonstrated adequate knowledge of the importance of long‐term breastfeeding, which, they said, helps children be healthy, as contained in the excerpt below.

Giving the breast milk is good, it helps the child to be well. All my children I breastfed them up to two years, this my child, for you to push her down, it would be difficult. When you allow the child to breastfeed up to the two years, it makes the child healthy. (IDI, mother of a four‐year‐old child, south zone)

It is not easy to breastfeed for two years, this my child, I had to stop him from feeding up at a point, it is not easy, I do some many things for us to get food to eat, I farm, I trade. so, I had to wean him off early in other to have time to work. (IDI, mother of a three‐year‐old child, North zone)

#### 3.3.3 Frequency of breastfeeding

Regarding the number of times a mother should breastfeed a child, the majority of the participants said breastfeeding cannot be counted since it is something that is done continually. Others also held the view that children are not the same, so their feeding pattern is not the same.

That question is hard…(Laughing) you can't count the number of times the child breastfeeds. That one you can't count, children are not the same, a child is there, doesn't sleep always breastfeeding. (IDI, mother of a four‐and‐a‐half‐year‐old child, south zone)

The common challenges of breastfeeding mentioned were sore nipples and waist pain as a result of sitting for long hours to breastfeed. Some participants also said it took an average of a day for the breast milk to flow, and within the period, they did not give anything to the baby.

The problem I can remember about breastfeeding was waist pains. Sitting all the time for the child to suck wasn't easy, it was painful but since you have the child, what can you do. (IDI, mother of a three‐year‐old child, north zone)

### 3.4 Knowledge about complementary feeding

The majority of the participants reported that, after 6 months, breastmilk alone could not meet the nutrient needs of infants. According to some of the mothers, the community health nurses have indicated “weanimix” or “koko” are nutritious complementary foods. These can be prepared using water, maize, or millet mixed with soya beans, groundnuts, and dried fish. They also mentioned mashed kenkey (a maize‐based food) with powdered milk, stock from vegetable soups, beans prepared with palm oil, and eggs as essential complementary feeds given to babies after 6 months. However, some had challenges getting fresh vegetables during the dry season. The following quotes illustrate the exact views presented by mothers on the issue:

You can give Koko, grind soya beans, groundnuts and Amani into flour and use this to prepare Koko for the child to drink when you go to the market, you can buy yoghurt for the child. but, (Sighs)In the dry season, it is not easy to get all the ingredients to prepare winimix because food items are very expensive and the vegetables too are just a few and they sell for five Ghana cedi's. (IDI, mother of a two‐year‐old child, north zone)

Another respondent said this:

When it is dry season, everything is very expensive but I have chickens when the chicken lay eggs, he will pick one of the eggs and give to me to cook for him. He doesn't joke with his eggs (laughing). (IDI, mother of a three‐year‐old child, south zone)

Some participants also said they started feeding these children regular adult staple food such as Tuo‐Zaafi (TZ), a local meal made from millet or maize and okro soup or “soro” (a type of slimy soup made from baobab leaves or false sesame leaves). The views expressed by the mothers suggested that the children's food was often prepared separately. Also, a few mothers said, when they first introduced complementary feeding, their children had diarrhea, which they attributed to their inability to maintain appropriate hygiene practices.

When you wake up and there aren't enough food ingredients, you have to prepare their food separate. If you want to prepare the food for the entire family, that is not the best their food is separate, you have to prepare the children food separate. (IDI, mother of a three‐year‐old child, south zone)

Another mother had this to say on the issue.

…how I introduced it, I was not careful, in terms of hygiene I was not careful, it was difficult, not that I wasn't hygienic but what you are supposed to do to preserve the food for instance porridge, how to wash the bottles well to prevent contamination, it was not properly done so the baby was sick. (IDI, mother of a four‐and‐a‐half‐year‐old child, north zone)

Also, the majority of the participants said children need much attention since they are growing up. So, for a mother, learning how to prepare different meals for children would entice them to eat. The following quote illustrates this:

A child needs attention, when you prepare this today, the next day you change the food. This helps the child to have an appetite but when you wake up and you prepare this and the child says she doesn't like, you don't just leave; you have to prepare something different. (IDI, mother of a two‐year‐old child, north zone)

### 3.5 Overcoming sociocultural practices/barriers

Most of the participants mentioned that there is a traditional rite in the communities where new‐born babies are made to drink herbal tea. The herbal tea is made from green herbs and roots, and its essence is to strengthen the bones of the newborn. However, the mothers said, they refused to allow their babies to drink the herbal teas. Some mothers expressed the challenges they went through after they allowed their children to drink the herbal tea.

Aside from the babies, nursing mothers are usually forced to take cultural baths. This is called “Sooru” in Kasem and “Kosoto” in Nankani. The process involves pouring warm herbal water over the mother for 3 days if the child is male and for 4 days if the child is female. The aim is to strengthen the nursing mother in order to help her feed her new‐born baby. Below are some of the excerpts from the interview.

It is because I disobeyed them, how I refused to perform the rites, it is by God s grace that nothing happened to the child, if something had happened to the child within that family, they would say it is because of my disobedience that led to the child's death, because I disobeyed them, in the name of Jesus Christ, God helped me, nothing happened. As at now, the child is alive, nothing has happened to the child. To be honest with you, it still happens, they are still doing it. Those cultural practices are still there. (IDI Mother of a three‐year‐old child, north zone).

I suffered a lot with my first child, she was always running diarrhea and passing out green stool, so when I delivered the second child, I told them I won't perform the rites again. They said I don't respect, they insulted me, they said I am a poor person, my husband married to a poor person and I don't respect people and I said yes, I accept that I am a poor person, I won't do that again, I won't perform the traditional rites. (IDI, mother of a three‐and‐a‐half‐year‐old child, north zone).

Aside from traditional practices, protein foods such as meat and eggs were not allowed to be given to children. The belief is that the early introduction of these kinds of foods was not good for children. Mangoes were also not suitable for children; the reason is that they could get malaria from eating them. According to the data, most of the mothers refused to comply with these cultural restrictions because they felt these protein foods would rather help their children grow well and be healthy. The following quotes illustrate these points.

…the old women and old men say that if you give an egg to your child, they say, you are teaching the child to be a thief, if you give meat, they say, you are trying to teach the child to be a thief, because of the little education I have, I don't agree to that, I know if I give an egg to my child, I'm doing that for my child to be well. If you give meat to a child, it is because you want the child to be well. You can't give birth to your child and be thinking that tomorrow, he /she will be a thief. Those who don't have education, when you do that, they think that you are teaching the child to be a thief. (IDI mother of a four‐year‐old child, south zone)

### 3.6 Support from mothers‐in‐law and elderly women

Some of the participants said that after delivery, their mother‐in‐law's and some elderly women in the household supported them. They prepared warm baths for them; some also said they helped to take care of their nutritional needs by preparing local meals and other beverages like “Zuumkum” for them to eat. Thus, the support of mother‐in‐laws promoted optimal infant feeding since mothers had enough time to breastfeed.

My husband's mother and older women in the house, after I delivered and came home, they helped me, they prepared “zuumkum” (a beverage made from guinea corn or white millet) she prepared that for me to drink, they added hot water to it for me to drink. In the afternoon, if I get tea I drink, if I don't get that I drink zuumkum, it helps in producing breastmilk. They also prepare TZ with soro for me to eat. They can use “Ayoyo” (green vegetable) or dry okro to prepare the soup, they add salt bitter to it, they believe salt bitter helps to heal the womb. (IDI, mother of a two‐year‐old child, south zone)

### 3.7 Receiving support from husbands

In terms of accessing health care, the majority of the participants mentioned that they could send their children to the health facility without waiting for the husband to grant permission. However, it is an act of courtesy to inform one's husband before doing anything. Most of them said their husbands know the importance of child welfare services, so would not prevent them from seeking health care. Also, from the views expressed by the mothers, it is realized that mother‐in‐laws usually want to exert their authority on the younger mothers, but due to their husband's support, this has often been averted. The following quote illustrates this:

If I say my husband doesn't support me then I'm telling lies, every day he baths and send them to school, he supports me a lot, I remember when they wanted to “Nyen” (feeding newborn with herbal tea) my child, my husband refused; it wasn't easy for us but he persisted for them not to “Nyen” the child. (IDI, mother of a three‐year‐old‐child, north zone)

## 4 DISCUSSION

The main aim of this present study is to find out what mothers/caregivers of well‐nourished children do to improve the nutritional status of their children. After data analyses, four important themes were observed from the data that explain the appropriate adoption of IYCF practices by the mothers. They include knowledge about breastfeeding (sub‐themes include initiation, duration, and frequency of breastfeeding), knowledge about complementary feeding, receiving support from husbands, mothers‐in‐law, and elderly women, and overcoming sociocultural barriers.

As recommended by the WHO, adequate knowledge on breastfeeding, such as early initiation of breastfeeding, breastfeeding through the age of 2 years and beyond, and the introduction of safe solid and semi‐solid foods at age 6 months is critical for early child survival (World Health Organization & United Nations Children's Fund (UNICEF), 2003). From the data, mothers are said to have initiated breastfeeding early and also breastfed for a long period of time. In addition, these mothers acknowledged the importance of feeding their children frequently. As stated by the WHO, after 6 months, breastmilk alone is not sufficient to meet the nutritional needs of infants (World Health Organization & United Nations Children's Fund (UNICEF), 2003). From the data, “weanimix” is mentioned as a major complementary feed available to babies. In our study, mothers reported that based on the teaching of community health nurses, they prepared “weanimix” at home as a complementary feed for the children during weaning. This finding is similar to a study carried out in the Ejura‐Sekyedumase district in the Ashanti Region of Ghana. The study shows that weanimix (a blend of beans, groundnuts and maize) is the most common complementary food given to infants in Ghana (Kumi et al., 2014).

Although mothers had knowledge of appropriate feeding practices, the challenge was inadequate food, which arises during the dry season. Therefore, improved agriculture to ensure food security is an important element to safeguard the availability of stable food in the area (Debpuur et al., 2021). Another mitigation measure is the use of dry‐season gardens and irrigation, which are still fraught with challenges such as improved agricultural inputs (Nonterah et al., 2022). The impact of this seasonable variation in the availability of food has been demonstrated to contribute to the occurrence of severe acute undernutrition (Nonterah et al., 2022). From the data, an egg was the most common protein product for children and women are practically involved in the rearing of chickens. This finding is similar to other study findings (Alders & Pym, 2009), where it is shown that poultry rearing plays an important role in poor rural households through the provision of scarce animal protein in the form of meat and eggs to enhance their diet and income.

It has been reported that the introduction of prelacteal feed to babies before they attain 6 months of age is a common practice in sub‐Sahara Africa (Berde & Ozcebe, 2017). Contrary to this, we observed that this was not a common practice in our study. This may be due to the extensive education given by the community health workers and volunteers based at Community‐Based Health Planning and Service Programme (CHPS) centers within the communities. These findings are similar to existing literature that suggests that health education programs relating to maternal and child health in rural communities contribute to colostrum feeding, early introduction of breastfeeding, and exclusive breastfeeding (Perez et al., 2009). Further supporting the education hypothesis is a study from northwest Ethiopia (Biks et al., 2015) that observed that mothers who had received antenatal care or had supervised delivery in a health institutional birth were more likely to practice exclusive breastfeeding.

We also found out that, culturally, infants and young children are not supposed to be fed eggs and meat since they perceive that such children will grow to be thieves in the future. This has been previously reported in a study that examined culture and community perceptions on diet for maternal and child health in the study area, which revealed that giving eggs to children will influence them to become thieves in the future (Dalaba et al., 2021). Similarly, this finding corroborates a previous study that reported on the cultural beliefs that prohibit the eating of eggs (Biks et al., 2015; Chege et al., 2015). Most of the older people believed that when children are fed eggs and meat, it predisposes them to steal from adults' life. This traditional belief has the potential to compromise appropriate child‐feeding as children are denied the nutrients provided by eggs and meat. It is therefore conclusive to say that these restrictions harm the nutritional status of children. It is therefore a positive direction that these mothers fed their children with protein products.

From the data, it is also realized that most of the grandmothers wanted to introduce early feeding to the infants, which is herbal tea. According to them, it is a cultural rite that has been practiced for several years, so it would be against their culture for the infants not to be fed the herbal teas. This finding is consistent with a study that was done in northern Ghana (Aborigo et al., 2012). However, in a systematic review, it was found that engaging family members, such as grandmothers, grandfathers, and other family members, can support IYCF practices and child survival, which is shown in the study findings (Martin et al., 2020).

In African society, grandmothers are respected individuals in households, and younger women are mandated to honor them. They are often considered to be the primary support providers for their daughters‐in‐law during pregnancy and after delivery. From the data, it was found that mothers‐in‐law were very supportive, and this helped the mothers to take care of their children. This finding is consistent with a study done in the Kassena‐Nankana District, which reported that mothers of husbands have a lot of power in households (Gupta et al., 2015).

Husbands act as major gatekeepers and primary decision‐makers within households in the African setting, effectively determining the care‐seeking practices of women during the perinatal and postnatal periods. According to the study, most of the mothers reported that they had support from their husbands, which enabled them to seek early health care for their children without waiting on them to grant permission. Aside from this, preventing mothers‐in‐law from initiating prelacteal feeding for the children contributed to their good health. This study finding is consistent with a study done in two rural sub‐districts of Bangladesh, which showed that, when husbands are involved in maternal health issues, it helps mothers to use skilled maternal health services, which translates into improving child survival (Rahman et al., 2018).

This study has not only shown the benefits of social support in promoting IYCF practices, but most importantly, it has shown that women go through a lot of challenges in promoting the general health of their children.

### 4.1 Strengths and limitations

Interviews were conducted in the local languages of the study area and translated into English for analysis. It is possible that the real meaning of some statements made in the local languages may have been lost in the English translation. Nevertheless, the interviews were translated and transcribed by experienced research assistants who are natives of the area, and hence the misinterpretation of the statements or words made in the interviews would be minimal and may not affect the results of the study. Also, the study participants had to delve into the past to provide information. This may have led to recall bias. However, appropriate probes were made to minimize this, which also helped to obtain suitable information.

In addition, the study team did not include children aged 6–23 months.

Despite these limitations, the study does identify key contextual factors that may serve as a point of reference for education and further improvements in IYCF practices. The study was scientifically sound as it employed appropriate sampling methods to avoid systematic sampling that may skew the views expressed. We ensured different layers of quality control to further strengthen the quality of the data and to also ensure data saturation was achieved. The study finally sets the stage for further studies to compare different practices between well‐nourished and malnourished children.

## 5 CONCLUSIONS

Based on the interpretations of our data, we observed that appropriate practices of IYCF among mothers contributed to the adequate nutritional status of their children. It also stood out strongly that while mothers adopted these practices, social and economic support from family and the community were likely to improve adoption, adherence, and sustainability of IYCF practices. Therefore, the inclusion of stakeholders such as family heads, mothers‐in‐law, community opinion leaders, and chiefs in program interventions aimed at improving nutrition will be a major boost to program fidelity and success.

## AUTHOR CONTRIBUTIONS

**Emmanuel Nakua:** Conceptualization (equal); writing – review and editing (equal). **Edith Dambayi:** Conceptualization (equal); resources (equal); writing – original draft (equal); writing – review and editing (equal). **Raymond A. Aborigo:** Conceptualization (equal); formal analysis (equal); methodology (equal); supervision (equal). **Samuel T. Chatio:** Supervision (equal); validation (equal); writing – review and editing (equal). **Maxwell A. Dalaba:** Writing – review and editing (equal). **Enos Sekwo:** Investigation (equal). **James K. Adoctor:** Investigation (equal). **Winfred Ofosu:** Supervision (equal). **Abraham R. Oduro:** Supervision (equal); writing – review and editing (equal). **Engelbert A. Nonterah:** Supervision (equal); validation (equal); writing – review and editing (equal).

## FUNDING INFORMATION

Edith Dambayi was supported by a scholarship from the INPreP project at the Navrongo Health Research Centre to undertake a masters in population and reproductive health at the Kwame Nkrumah University of Science and Technology (KNUST). The broader INPreP study is supported by the National Institute for Health Research (NIHR) under grant/award number 17\63\154 using UK aid from the UK Government to support global health research. The views expressed in this publication are those of the author(s) and not necessarily those of the NIHR or the UK Department of Health and Social Care.

## CONFLICT OF INTEREST STATEMENT

The authors declare no conflicts of interest.

## Data Availability

The data that support the findings of this study are available from the corresponding authors upon reasonable request.
